# Nephroprotective Effects of Tetramethylpyrazine Nitrone TBN in Diabetic Kidney Disease

**DOI:** 10.3389/fphar.2021.680336

**Published:** 2021-06-24

**Authors:** Mei Jing, Yun Cen, Fangfang Gao, Ting Wang, Jinxin Jiang, Qianqian Jian, Liangmiao Wu, Baojian Guo, Fangcheng Luo, Gaoxiao Zhang, Ying Wang, Lipeng Xu, Zaijun Zhang, Yewei Sun, Yuqiang Wang

**Affiliations:** ^1^ Institute of New Drug Research, International Cooperative Laboratory of Traditional Chinese Medicine Modernization and Innovative Drug Development of Chinese Ministry of Education, Jinan University College of Pharmacy, Jinan University, Guangzhou, China; ^2^ Department of Gerontology, The First Affiliated Hospital of Jinan University, Guangzhou, China; ^3^ Department of Neurology, The First Affiliated Hospital of Jinan University, Guangzhou, China; ^4^ Institute of Chinese Medical Sciences and State Key Laboratory of Quality Research in Chinese Medicine, University of Macau, Avenida da Universidade, Taipa, Macao

**Keywords:** diabetic kidney disease, nephroprotection, metabolic abnormalities, oxidative stress, mitochondrial function

## Abstract

Diabetic kidney disease (DKD) is the leading cause of end-stage renal failure, but therapeutic options for nephroprotection are limited. Oxidative stress plays a key role in the pathogenesis of DKD. Our previous studies demonstrated that tetramethylpyrazine nitrone (TBN), a novel nitrone derivative of tetramethylpyrazine with potent free radical-scavenging activity, exerted multifunctional neuroprotection in neurological diseases. However, the effect of TBN on DKD and its underlying mechanisms of action are not yet clear. Herein, we performed streptozotocin-induced rat models of DKD and found that TBN administrated orally twice daily for 6 weeks significantly lowered urinary albumin, N-acetyl-β-D-glycosaminidase, cystatin C, malonaldehyde, and 8-hydroxy-2′-deoxyguanosine levels. TBN also ameliorated renal histopathological changes. More importantly, in a nonhuman primate model of spontaneous stage III DKD, TBN increased the estimated glomerular filtration rate, decreased serum 3-nitrotyrosine, malonaldehyde and 8-hydroxy-2′-deoxyguanosine levels, and improved metabolic abnormalities. In HK-2 cells, TBN increased glycolytic and mitochondrial functions. The protective mechanism of TBN might involve the activation of AMPK/PGC-1α-mediated downstream signaling pathways, thereby improving mitochondrial function and reducing oxidative stress in the kidneys of DKD rodent models. These results support the clinical development of TBN for the treatment of DKD.

## Introduction

Diabetic kidney disease (DKD) is one of the leading causes of end-stage renal disease worldwide, on top of being a major risk factor for both cardiovascular diseases and death (Umanath and Lewis, 2018). The *International Diabetes Federation Diabetes Atlas* (ninth edition) reports that the number of diabetic patients is projected to rise from 463 million to 700 million adults worldwide by 2045 (Huang et al., 2018). Approximately one-third of subjects with type 1 diabetes and half of individuals with type 2 diabetics develop DKD (Reutens, 2013; Koye et al., 2018). As the incidence of diabetes rises, so will the incidence of DKD, exacerbating what is already a significant health and economic burden on society.

Current strategies for treating DKD focus primarily on systemic intervention for diabetes-related metabolic changes (i.e., hyperglycemia, hypertension, and dyslipidemia). Moreover, clinical guidelines worldwide recommend renin-angiotensin-aldosterone system (RAAS) blockade as first line therapy for DKD. However, current DKD therapeutics do not prevent or reverse renal diseases from progressing to end stage (Zac-Varghese and Winocour, 2018). Most recently, sodium glucose transporter-2 inhibitors (SGLT2i), a new class of anti-hyperglycemic agents, have come on the scene as potential drug candidates for DKD. Treatment with SGLT2i increases the risk of genital infections and bone fractures (Kohan et al., 2014; Watts et al., 2016). In addition, the U.S. Food and Drug Administration (FDA) recently strengthened their warning that SGLT2i agent canagliflozin and dapagliflozin may increase the risk of acute kidney injury and failure (Administration, 2016). The options for effective and safe treatment of DKD are therefore still limited, making the necessity of developing novel therapies a more pressing issue than ever.

The pathogenesis of DKD is complex and manifests itself in the impairment of both glomerular and tubular function. Although the precise etiology of DKD remains unclear, it is clear that hyperglycemia-induced oxidative damage to mitochondrial constituents (DNA, proteins, and lipids) results in mitochondrial dysfunction in DKD (Forbes and Thorburn, 2018). Circumstantial evidence supporting the involvement of mitochondrial dysfunction in DKD has been accumulating from animal and *in-vitro* studies (Sun et al., 2008; Nusinovici et al., 2019). Recent studies have directly demonstrated that mitochondrial dysfunction is present in clinical DKD samples: for example, Czajka et al. reported that mitochondrial DNA and mitochondrial RNAs are altered in the blood cells of DKD patients (Czajka et al., 2015). Furthermore, end-stage mitochondrial dysfunction can lead to intrinsic cell death (Murphy, 2009; Forbes and Thorburn, 2018). Therapeutic interventions for DKD must thus incorporate strategies directed towards interrupting the mitochondrial production of reactive oxygen species (ROS), alleviating mitochondrial dysfunction, and inhibiting downstream deleterious pathways.

AMP-activated protein kinase (AMPK) is a heterotrimeric serine/threonine protein kinase that serves as a cellular energy regulator. AMPK is tightly regulated by the AMP/ATP ratio and it is activated under high AMP-to-ATP conditions (Herzig and Shaw, 2018). Recent studies have suggested that there is a loss of AMPK activity in DKD and that AMPK activation improves DKD by inducing mitochondrial biogenesis and antioxidant defense systems (Akhtar and Siragy, 2019). The master regulator of mitochondrial biogenesis is peroxisome proliferator-activated receptor-gamma coactivator-1α (PGC-1α); once activated through either phosphorylation or de-acetylation, PGC-1α translocate into the nucleus where it can bind to and activate nuclear respiratory factors 1 and 2 (Nrf1/Nrf2). Subsequently, Nrf1 and Nrf2 activate mitochondrial transcription factor A (TFAM) and bind to promoter regions of nuclear coded respiratory chain components, thereby increasing mitochondrial DNA replication and transcription, the synthesis of mitochondrial proteins, and mitochondrial biogenesis (Fontecha-Barriuso et al., 2020). Moreover, transcriptional regulation of PGC-1α is responsible for the up-regulation of the Nrf2-antioxidant response element pathway in response to ROS produced by mitochondrial dysfunction (Li and Susztak, 2018). Overall, AMPK activation improves DKD by increasing PGC-1α-regulated mitochondrial biogenesis and nuclear factor Nrf2-induced downstream antioxidant defense.

Tetramethylpyrazine (TMP, Supplemental Figure 1) is an active ingredient of the herbal medicine *Ligusticum wallichii* Franchat (Guo et al., 2016; Wang et al., 2017). TMP has been well documented to scavenge ROS (Zhang et al., 1994). Previous studies have shown that TMP can alleviate kidney damage induced by ischemia/perfusion in rats *via* scavenging oxygen free radicals (Feng et al., 2004). A meta-analysis of the clinical effect of TMP on DKD suggests that injected TMP significantly improves renal function in DKD patients (Wang et al., 2012). To enhance the antioxidant activity and protective effects of TMP, we designed and synthesized a novel derivative of TMP, 2-[[(1,1-dimethylethyl)-oxidoimino]-methyl]-3,5,6-trimethylpyrazine (TBN, Supplemental Figure 1), which contains a powerful free radical scavenging nitrone moiety (Sun et al., 2008). The nitrone group is the active pharmacophore of disodium 2,4-disulphophenyl-N-tert-butylnitrone (NXY-059), once the subject of intense clinical investigation (Lees et al., 2006). In our previous study, we demonstrated that TBN has potent free radical scavenging activity against some of the most damaging radicals, including hydroxyl, superoxide, and peroxynitrite. Furthermore, it protects mitochondrial function and prevents neuronal damage in primary cortical neurons (Zhang et al., 2018). These findings suggest that TBN may have the potential to reduce the severity of oxidative stress injury and mitochondrial dysfunction in DKD.

In the current study, we examined the therapeutic effects of TBN in three different experimental models of DKD, and further elucidated its mechanisms of action for nephroprotection.

## Materials and Methods

### Reagents and Materials

Streptozotocin (STZ), DCFH-DA, and ADP/ATP Ratio Assay kit were purchased from Sigma-Aldrich (Kansas, MO, United States). MitoSOX Red was purchased from Invitrogen (Carlsbad, CA, United States). XF Cell Mito Stress and XF Glycolysis Stress Test Kits were purchased from Agilent (Santa Clara, CA, United States). Bicinchoninic acid (BCA) protein assay kit and JC-1 probe were bought from Beyotime Institute of Biotechnology (Shanghai, China). Enhanced chemiluminescence (ECL) kit was purchased from Fude biological technology Co., Ltd. (Hangzhou, China). Monkey 8-OHdG ELISA kit (ab201734) was purchased from Abcam (Cambridge, United Kingdom). Erythropoietin (EPO), 3-nitrotyrosine (3-NT) and glutathione peroxidase seven activity (GPx7) ELISA kits and malondialdehyde (MDA) assay kit were purchased from Nanjing Jiancheng Bioengineering Institute (Nanjing, China).

The following primary antibodies were used: TGF-β1 antibody (ab92486), Col-IV (ab6586), Nrf1 (ab34682), and TFAM (ab131607) were purchased from Abcam (Cambridge, United Kingdom). Brn3a (sc-31984) and IgG-FITC (sc-2024) were purchased from Santa Cruz Biotechnology (Santa Cruz, CA, United States). Insulin (4,590), phosphor-AMPK (*p*-AMPK, 2,535), total AMPK (5,831), GAPDH (5,174), and secondary antibodies (7,074 and 7,076) were all from Cell Signaling Technology (Danvers, MA, United States). PGC-1α (NBP1-04676), Nrf2 (NBP1-32822), and HO-1 (NBP1-31341) were bought from Novus (Littleton, Colorado, United States).

### Animals and Experimental Schedule

Male and female Sprague-Dawley (SD, 180–200 g) rats were purchased from Guangdong Medical Laboratory Animal Center (Guangdong, China). The rats were housed with a 12 h light/dark cycle and free access to food and water in a temperature of 20–25°C and humidity of 30–50%. Procedures were approved and supervised by the Institutional Animal Care and Use Committee of Guangzhou University of Chinese Medicine. Diabetes was induced in SD rats via a single intraperitoneal injection of 55 mg/kg STZ, freshly dissolved in 0.1 M citrate buffer (pH 4.5). The control animals were given a single intraperitoneal injection with an equal volume of citrate buffer. Rats with blood glucose levels ≥16.7 mmol/L at 3 weeks post-STZ injection were included in this study. Next, STZ-induced diabetic rats were treated with TBN (10, 30, or 60 mg/kg body weight, twice daily) or the positive control losartan (10 mg/kg body weight, once daily) for 6 weeks.

Male rhesus monkeys 14–22 years of age with spontaneous DKD were obtained from Sichuan PriMed Shines Bio-Tech Co., Ltd. (Sichuan, China). The studies were conducted according to guidelines from the Experimental Animal Care and Use Committee of Sichuan PriMed Shines Bio-Tech Co., Ltd. The experimental protocols were approved by the Ethics Committee for Animal Experiments of Sichuan PriMed Shines Bio-Tech Co., Ltd. The selected rhesus monkeys with stage III DKD met the following criteria: estimated glomerular filtration rate (eGFR) = 30–59 ml/min/1.73 m^2^, cystatin C (CysC) ≥ 1.3 mg/dl, serum creatinine (Scr) ≥ 1.26 mg/dl, weight stable, and not yet receiving insulin or any other therapy. Seven rhesus monkeys in total were assigned to the vehicle (*n* = 2) and TBN groups (*n* = 5). TBN was administered to rhesus monkeys as a subcutaneous administration of 30 mg/kg twice daily for 12 weeks.

### Metabolic Measurements

In the rat studies, body weight was measured weekly. After 6 weeks of treatment, the rats were placed in individual metabolic cages to collect 24 h urine for urinary protein, albumin, and urinary N-acetyl-β-D-glycosaminidase (NAG) analysis. At the time that the rats were sacrificed, blood and tissue samples were harvested and processed for various studies. Blood glucose, Scr, urea nitrogen (BUN), total cholesterol (TC), triglycerides (TG), NAG, CysC and serum iron were measured by an automatic biochemical analyzer (Hitachi Auto Analyzer 7,100, Hitachi Co. Ltd., Tokyo, Japan). The kidneys and pancreases were excised for histological analysis. A portion of the renal cortex was reserved for biochemical and ultrastructural analysis.

In the rhesus monkey study, body weights were measured weekly throughout the course of the study. Blood samples were taken by venipuncture at 0, 4, 8, and 12 weeks for biochemical measurements. Plasma creatinine (Cr-P), CysC, HbA1c, TG, TC, and low-density lipoprotein (LDL) were measured using an automatic biochemical analyzer (Roche cobas6000 analyzer series C501). Renal function was evaluated using the clinically relevant surrogate markers, Cr-P, CysC concentrations, as well as eGFR. eGFRmale = 135× min (Cr-P/0.9,1)−0.207 × max (Cr-P/0.9,1) 0.601 × min (CysC/0.8,1) 0.375 × max (CysC/0.8,1) 0.711 × 0.995 (Age×3), and is a measurement commonly used in clinic to measure kidney function. Intravenous blood samples were also taken for biochemistry measurements done by a clinical analyzer.

### Histopathology

Renal tissue samples were harvested for pathologic examination. Tissues were fixed in 4% paraformaldehyde overnight, embedded in paraffin and processed for sectioning. After deparaffinization and rehydration, the paraffin sections (4 µm) were stained with hematoxylin and eosin (H&E), periodic acid-Schiff (PAS), and Masson, respectively. Glomerular size was determined using ImageJ image analysis software. Glomerulosclerosis was defined as the percentage of extracellular matrix deposition. Extracellular matrix deposition in glomeruli was evaluated by PAS staining, whereby the percentage of mesangial matrix occupying each glomerulus was rated on a scale from 0 to 4 as follows: 0, no glomerulosclerosis; 1, sclerosis in <25% of the glomerulus; 2, sclerosis in 25–50% of the glomerulus; and 3, sclerosis in 50–75% of the glomerulus; 4, sclerosis in >75% of the glomerulus. Interstitial fibrosis was graded on a scale of 0–4 on Masson-stained renal tissues. The semiquantitative scoring was as follows: 0, no fibrosis; 1, fibrosis <10% of areas; 2, fibrosis 10–25% of areas; 3, fibrosis 25–50% of areas; and 4, fibrosis >50% of areas. Retinal tissue from SD rats was cut into 4 µm sections for H&E staining. Retinal sections were imaged to analyze the thickness of each retinal layer, including the ganglion cell layer (GCL), internal plexiform layers (IPL), inner nuclear layer (INL), and outer nuclear layers (ONL). Images were captured using a light microscope (Olympus, Japan). The thickness of each retinal layer was quantified using ImageJ software (National Institutes of Health, Bethesda, MD, United States). The pathologic changes were assessed by four researchers who were blinded to this study.

### Immunohistochemistry

Kidneys were fixed in 4% paraformaldehyde overnight, embedded in paraffin and processed for sectioning. After deparaffinization and rehydration, the kidney tissue sections (4 µm) were treated with 3% H_2_O_2_ for 10 min and with 1% bovine serum albumin in PBS for 30 min, then incubated overnight at 4°C with anti-TGF-β1 antibody (1:200), anti-Col-IV antibody (1:100), anti-MCP-1 antibody (1:100), anti-α-SMA antibody (1:100), and anti-Insulin antibody (1:100). Immunoreactions were detected using peroxidase conjugated anti-rabbit polymers and the DAB substrate kit (Gene Tech, China). Images were taken with a light microscope (Olympus, Japan). Samples were evaluated semiquantitatively from 30 randomized nonoverlapping fields using Image-Pro Plus software (Media Cybernetics, Inc., Rockville, United States).

### Immunofluorescence

Brn3a is a specific nuclear marker for retinal ganglion cells (RGCs). Retinas were incubated overnight with goat anti-Brn3a antibody (1:500) after permeation. After washing in PBS, retinas were incubated at room temperature for 1 h with donkey anti-goat IgG-FITC (1:500). After washing in PBS, retinas were mounted vitreal side up on slides and covered with antifading solution. Images were captured using an inversion fluorescence microscope (Olympus, Japan). Retinal cell numbers in the GCL were counted using Image-Pro Plus 6.0 (Media Cybernetics, Inc., Rockville, MD, United States).

### Enzyme-Linked Immunoassays

In rat studies, the final 24 h urine sample was collected from metabolic caging for albumin and 8-hydroxy-2′-deoxyguanosine (8-OHdG) analysis was done using a specific ELISA Kit after 6 weeks of drug or vehicle treatment. For insulin determination, the sera from SD rats were assayed using a rat insulin ELISA kit. For EPO determination, the sera from SD rats were assayed using rat EPO ELISA Kits, respectively. In the rhesus monkey study, blood samples were collected and centrifuged for 10 min at 3000 g, after which the sera was collected by aspiration. For 8-OHdG determination, the sera were assayed using a Monkey 8-OHdG ELISA kit. The concentrations of 3-nitrotyrosine (3-NT) and glutathione peroxidase seven activity (GPx7) in the rhesus monkey sera were measured using the Monkey 3-NT and Monkey GPx7 ELISA Kits, respectively. Those biomarkers were assayed using an ELISA Kit in accordance to the manufacturer’s protocol, and absorbance was measured at 450 nm.

### Malondialdehyde Measurement

The serum Malondialdehyde (MDA) levels were measured using commercially available assay kits. According to the manufacturer’s instructions, the MDA levels were measured by acid hydrolysis of the tetraethoxypropane method. The absorbance was measured at 532 nm.

### Tetramethylpyrazine Nitrone Concentrations in Plasma and Kidney Samples From Rats

Male and female SD rats (206–376 g) were purchased from Vital River Laboratory Animal Technology Co., Ltd. (Beijing, China). Animals were housed in standard cages on a 12 h cycle of light and darkness at an average room temperature range from 20 to 25°C and 40–70% humidity. All animals were allowed free access to food and water, and were fasted over 12 h before administration of TBN. The animal studies were conducted according to guidelines from the Experimental animal care and Use Committee of 3D BioOptima Co., Ltd. The experimental protocols were approved by the Ethics Committee for Animal Experiments of 3D BioOptima Co., Ltd. The pharmacokinetics studies were carried out by contract research organization (3D BioOptima Co., Ltd., Suzhou Ace Park, Jiangsu, China). In the non-clinical pharmacokinetics study, 18 rats were randomly divided into three groups, each group consisting of either sex. TBN was freshly prepared by dissolving the compounds in DMSO to obtain clear solution. Six rats were sacrificed at 10 min, 30 min, and 4 h after dosing of TBN (30 mg/kg), respectively. Blood and tissue samples were collected for TBN content detection by validated high-performance liquid chromatography-tandem mass spectrometry (HPLC-MS/MS) methods as previously described (Sun et al., 2014).

### The Pharmacokinetics of Tetramethylpyrazine Nitrone Tablets in Healthy Chinese Volunteers

The multiple-ascending-dose phase I study of TBN in healthy Chinese volunteers has been registered at http://www.chinadrugtrials.org.cn/(CTR20190583) and conducted in accordance with the Good Clinical Practices and the Declaration of National Medical Products Administration of China. The phase I was an open study testing ascending doses and pharmacokinetics in healthy Chinese volunteers. The study enrolled men and women aged 18–45 years with a body mass index of 19.5–26.0 kg/m^2^. Twenty-four healthy Chinese volunteers were divided into two groups, one of which received 600 mg of TBN (*n* = 12, male: female = 1:1), the other 1,200 mg of TBN (*n* = 12, male: female = 1:1). Subjects were given the desired amount of TBN in tablet form twice daily (interval 12 h) for seven consecutive days. Blood samples were collected in blood collection tubes with EDTA, centrifuged at 1,500 rpm for 10 min, and the supernatant transferred into labeled tubes before freezing at −80 ± 10°C until analysis. TBN’s concentrations in plasma were measured by HPLC-MS/MS (Waters, Massachusetts, United States).

### Cell Culture

HK-2 cells were a gift from Zhiqiang Ye (Fujian Institute of Clinical Geriatrics). HK-2 cells were maintained at 37°C in an atmosphere containing 5% CO2 in Dulbecco’s modified Eagle’s medium (DMEM)/Ham’s F12 medium (Invitrogen) supplemented with 10% fetal bovine serum (Gibco, Australia).

### Mitochondrial Glucose Flux and Glycolysis

Mitochondrial glucose flux and glycolysis were measured according to manufacturer’s protocol (Agilent Cell Analysis Technology) using the Seahorse XF96 Extracellular Flux Analyzer (Agilent, Santa Clara, CA). HK-2 cells were incubated with or without TBN (30 μM) for 24 h. Oxygen consumption rate (OCR) was measured using the XF Cell Mito Stress Test Kit (#103015-100, Seahorse Biosciences). Extracellular acidification rate (ECAR) was measured using the XF Glycolysis Stress Test Kit (#103020-100, Seahorse Biosciences). After baseline measurements, substrates or inhibitors of interests were injected at working concentrations of glucose (10 mM), oligomycin (1 µM), FCCP (0.5 µM), 2-DG (50 mM) and rotenone and antimycin A (0.5 µM). The respiratory rate was measured at 37°C and analyzed. The experiments were repeated 3 times. For each experiment, the means from 10 replicate wells were recorded.

### Assessment of Intracellular ROS Level and mtROS

The intracellular ROS and mtROS were determined using DCFH-DA and MitoSOX Red, respectively. HK-2 cells were plated in 96-well plates and incubated 24 h at 37°C. HK-2 cells were incubated with or without TBN for 48 h. DCFH-DA (10 µM) or MitoSOX Red (5 µM) was added to the cells and cultured for 20 min at 37°C. Following washing with PBS, the fluorescent intensity of the stained cells was detected using a multifunctional microplate reader.

### Mitochondrial Membrane Potential Assays

MMP was determined using the JC-1 probe according to the manufacturer’s protocol. Briefly, HK-2 cells were incubated with the JC-1 for 20 min at 37°C. Following washing with PBS, the “red” (excitation 525 nm, emission 590 nm) and “green” (excitation 490 nm, emission 530 nm) fluorescence were measured using a Fluorescence microplate reader (BioTek Instruments, Winooski, VT, United States). Mitochondrial depolarization (i.e., loss of MMP) manifests itself by a decrease in the red/green fluorescence ratio.

### The ADP/ATP Ratio Assay

The ADP/ATP ratio was measured with an ADP/ATP Ratio Assay kit according to the manufacturer's instructions. Briefly, the cells (10,000/well) were seeded in a 96-well, flat-bottom, white plate with clear bottoms. After removing the culture medium, 90 µL of ATP reagent was added and incubated for 1 min. The ATP level was measured using a multifunctional microplate reader and expressed as the number of relative light units (RLU). After 10 min, the ADP in the solution was converted to the ATP by adding of ADP converting enzyme (5 μL), and the RLU was measured immediately before and after 1 min of conversion. Subsequently, the ADP/ATP ratio was calculated as (C-B)/A, where A is the RLU for ATP, B is the RLU of ATP immediately before conversion from ADP, and C is the RLU of ATP at 1 min after conversion from ADP.

### Cataract Examination and Scoring

Cataracts were scored every week beginning at 3 weeks post STZ treatment. Following general anesthesia with isoflurane and pupil dilation with tropicamide (0.5% tropicamide and 0.5% phenylephrine; Xingqi Pharmaceutical, Shenyang, China), images of the rat lens were obtained with a Canon camera (Canon EOS 1100D, China) connected to a stereomicroscope (XTL-165, Phenix, China) using a digital camera microscope adapter (G2540, Cossim, China). Cataracts were scored according to the formation and progression of lenticular opacity as follows: 0: clear normal lens; 1: peripheral vesicles of lens; 2: peripheral vesicles and cortical opacities; 3: diffuse central opacities; 4: mature nuclear cataract.

### Western Blotting

Kidney tissue was harvested and lysed with RIPA buffer containing phosphatase and protease inhibitors, and 20 mg of total protein was subjected to SDS-PAGE analysis. After protein transfer to nitrocellulose membranes (Millipore; Billerica, MA, United States), the membranes were blocked with 5% skim milk for 1 h at room temperature and then probed with primary antibodies against phosphor-AMPK (1:1,000), total AMPK (1:1,000), PGC-1α (1:2,000), Nrf2 (1:1,000), HO-1 (1:1,000), and GAPDH (1:2,000) at 4°C overnight. After washing, the membranes were then detected by incubating with secondary antibody (1:2,000) conjugated with horseradish peroxidase and visualized using ECL Western blot detection reagents. All Western blots were repeated at least 6 times. The density of the immunoreactive membranes was quantified using Carestream molecular imaging system (Carestream Health, Inc., United States).

### Statistical Analyses

Statistics were performed using Prism seven statistics software (GraphPad Software). The experimental data were expressed as the mean ± SEM. A Student’s t test was used when comparing two groups. Multiple-group comparisons were evaluated using 1-way ANOVA or 2-way ANOVA followed by tukey’s test, as appropriate. *p* < 0.05 was considered statistically significant.

## Results

### Tetramethylpyrazine Nitrone Prevents the Progression of DKD in STZ-Treated Rats

**FIGURE 1:**
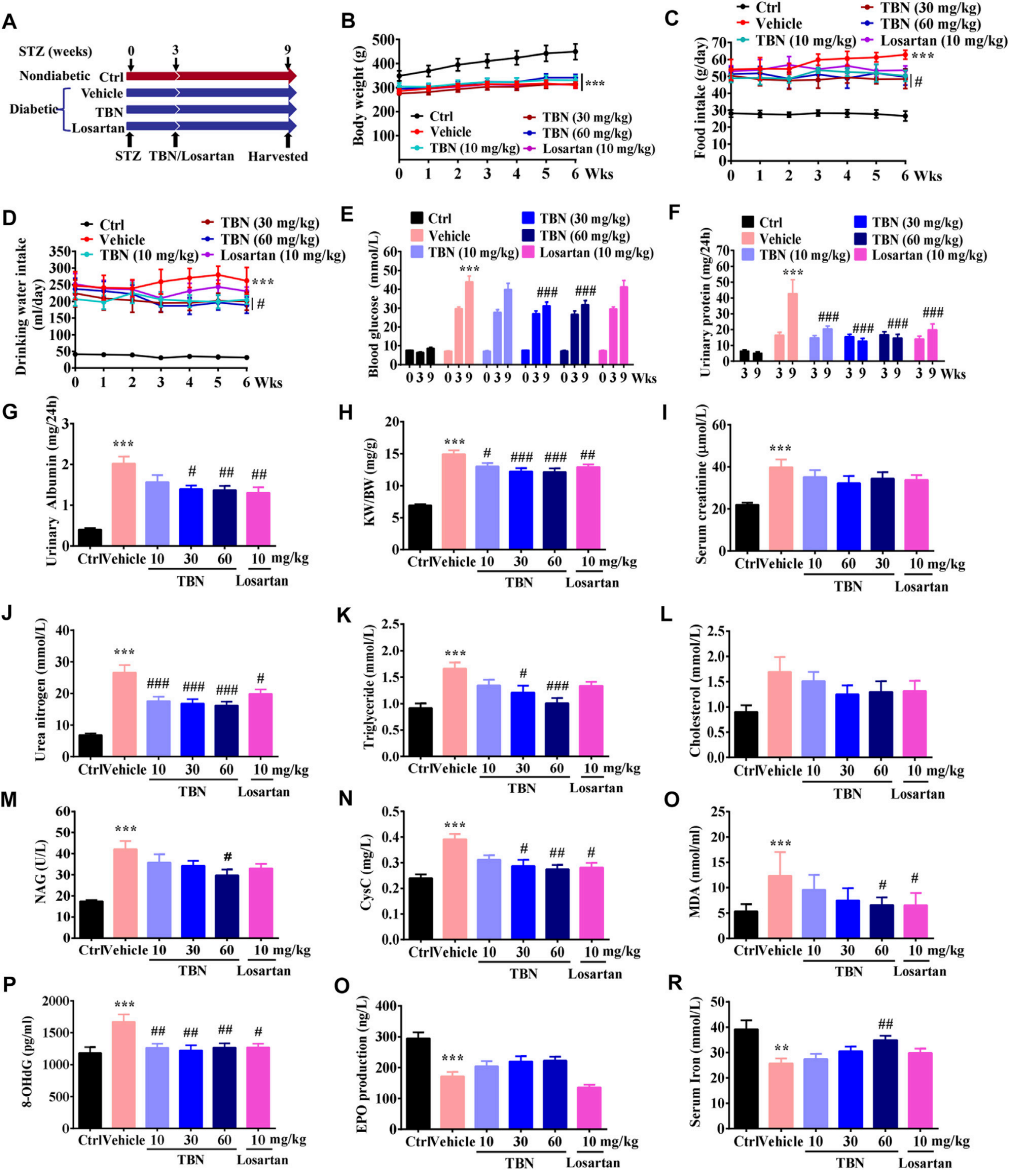
TBN prevents DKD in STZ-induced rats. **(A)** TBN or losartan treatment protocol in STZ-induced DKD rats. **(B)** Body weight, **(C)** food intake, **(D)** drinking water, **(E)** blood glucose level, and **(F)** urinary protein were measured 3 weeks post-STZ injection and 6 weeks post-drug treatment. **(G)** urinary albuminuria, **(H)** KW/BW, **(I)** serum creatinine, **(J)** serum urea nitrogen, **(K)** serum triglycerides, **(L)** serum cholesterol, **(M)** NAG, **(N)** CysC, **(O)** MDA, **(P)** 8-OHdG, **(Q)** EPO, and **(R)** serum iron were measured after 6 weeks of drug treatment. All data were shown as the mean ± SEM. Significance was determined by 2-way ANOVA in B-F or 1-way ANOVA in G-R followed by Tukey’s test. ** *p* < 0.01 and *** *p* < 0.001 vs. Ctrl group. ^#^ *p* < 0.05, ^##^ *p* < 0.01, and ^###^ *p* < 0.001 vs. vehicle-treated group.

STZ-induced type 1 diabetes has been widely used as a model for DKD (Ge et al., 2016). TBN was given twice daily orally for 6 weeks starting 3 weeks after STZ injection (Figure 1A). At the end of the experiments, body weights were significantly decreased in STZ-induced DKD rats when compared with age-matched normal control rats. There was no obvious difference in body weight between the vehicle-treated STZ group and the TBN- or losartan-treated groups (Figure 1B). Food intake, water consumption, blood glucose, urinary protein, and urinary albumin levels of experimental rats were significantly higher 3 weeks post-STZ injection as compared with normal control rats (Figures 1C-G). Food intake, water consumption, blood glucose, urinary protein, and urinary albumin levels were significantly decreased in the TBN treatment group (rats treated with TBN for 6 weeks) when compared to the STZ-induced DKD rats (Figures 1C–G). In contrast, rats treated with losartan had a reduction in only urine protein and urinary albumin levels (Figures 1F,G). Treatment with TBN and losartan significantly reduced the elevated kidney weight/body weight (KW/BW) in STZ-induced DKD rats (Figure 1H).

We then examined the effects of TBN on renal function in DKD rats; we found that urea nitrogen induced by STZ were all significantly reduced after treatment with TBN and losartan (Figure 1J), but not the serum creatinine (Figure 1I). Abnormal lipid metabolism is another hallmark of DKD. TBN treatment significantly lowered levels of blood triglycerides (Figure 1K) but not of cholesterol in STZ rats (Figure 1L). NAG, an early proximal tubular damage marker for the onset of DKD, is known to increase with hyperglycemia, even in normo-albuminuria conditions (Hong N. et al., 2018). Urinary excretion of CysC suggests tubular injury as it is elevated in early stages of diabetes and DKD (Garg et al., 2015; Uwaezuoke, 2017). We observed that both NAG and CysC were significantly increased in vehicle-treated STZ rats, and these increases were attenuated by either TBN treatment (Figures 1M,N). Losartan treatment also significantly decreased CysC levels (Figure 1N), but not the NAG (Figure 1M). These results indicate that TBN ameliorates the abnormalities in renal function caused by DKD in STZ-induced rats.

Oxidative damage includes lipid peroxidation and oxidative DNA damage. MDA is the stable metabolite of lipid peroxidation and has been extensively measured as an indicator for oxidative damage (Sharebiani et al., 2020). 8-OHdG is known to be a sensitive biomarker of oxidative DNA damage in urine and leukocytes (Liu et al., 2020). Either TBN or losartan treatment markedly decreased the levels of MDA and 8-OHdG when compared to that in the vehicle-treated STZ group (Figures 1O,P). These findings suggest that systemic oxidative damage, which was observed in DKD rats, was ameliorated by the antioxidant activity of TBN.

The incidence of anemia in DKD is clearly associated with the degree of albuminuria and decreased renal function. Correction of anemia thus improves quality of life and may delay the progression of DKD. Iron and EPO deficiency are the most common causes of renal anemia, and thus treatment is primarily targeted at these two conditions (Tsai and Tarng, 2019). Our results establish that vehicle-treated STZ rats displayed significantly lower amounts of EPO and serum iron, whereas TBN treatment increased both EPO and iron (Figures 1Q,R). By contrast, losartan slightly decreased EPO levels (Figure 1Q) and had no obvious effect on iron when compared with vehicle-treated STZ rats (Figure 1R).

### Tetramethylpyrazine Nitrone Reduces Pathological Changes in Renal and Pancreatic Tissue in STZ-Treated Rats

**FIGURE 2:**
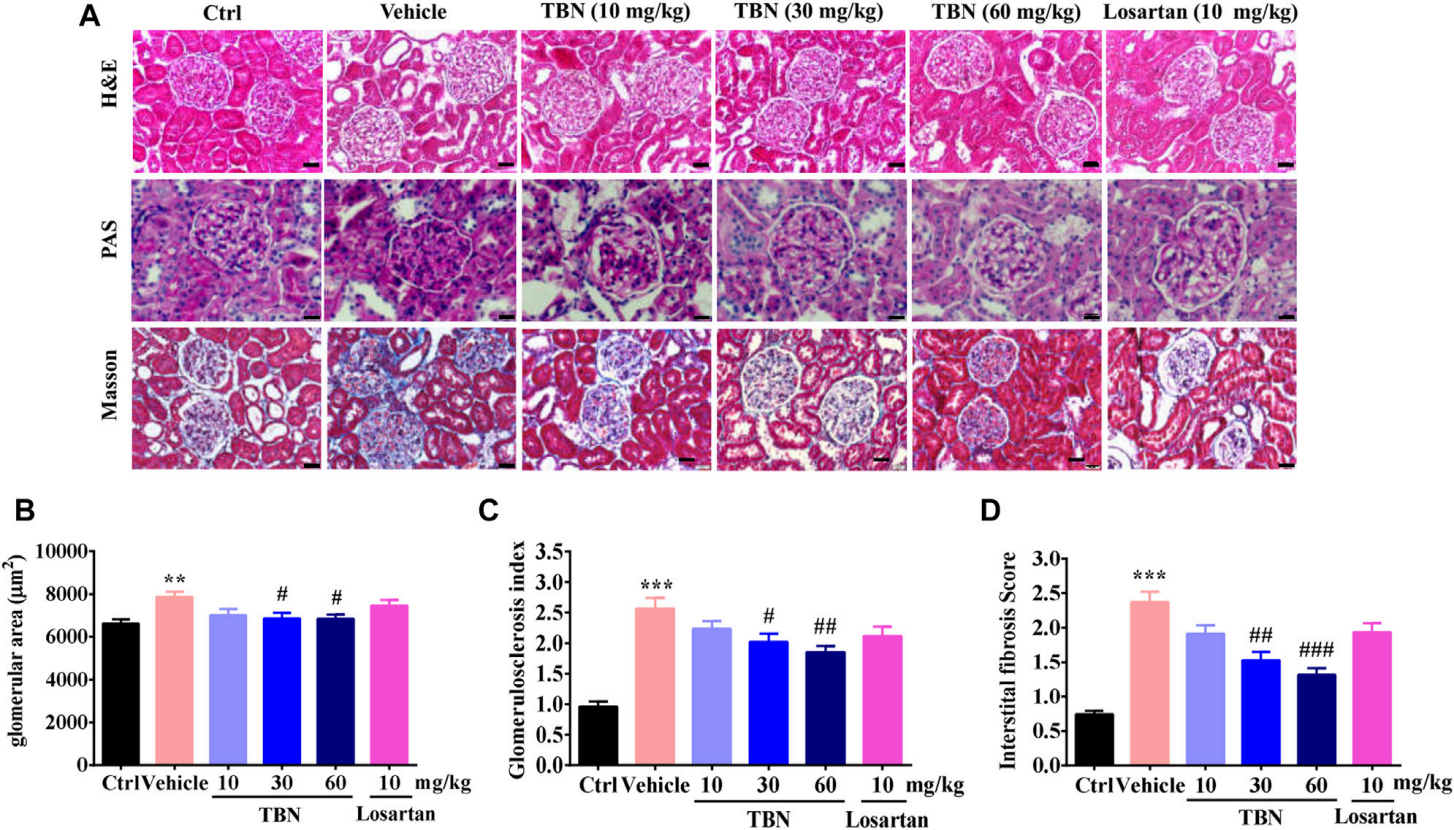
TBN reduces glomerular pathological changes in STZ-induced rats. **(A)** Representative photomicrographs of H&E staining, PAS staining, and Masson staining in the kidney. Scale bars, 50 μm in H&E and PAS staining, and 20 μm in Masson staining. (**B-D**) Semi-quantitative analysis of glomerular surface area, glomerulosclerotic index and tubulointerstitial fibrosis index were shown. 30 images of H&E, PAS, and Masson-stained kidney sections were counted for each rat. *n* = 13 for vehicle-treated DM group and *n* = 16 for other groups.

The pathological changes in STZ-induced DKD include glomerular hypertrophy, GBM thickening, mesangial expansion, glomerulosclerosis, and tubulointerstitial fibrosis (Ge et al., 2016; Liang et al., 2018). Figure 2A shows representative photomicrographs of H&E, PAS, and Masson trichrome staining in the kidney. H&E staining revealed obvious glomerular hypertrophy and mesangial cell proliferation in vehicle-treated STZ rats compared with control rats. PAS staining of renal cortex sections revealed increased mesangial expansion in vehicle-treated STZ rats compared with control rats. Masson staining further indicated glomerulosclerosis. TBN treatment significantly ameliorated these changes induced by STZ. Although mesangial matrix expansion, glomerulosclerosis, and interstitial fibrosis were observed to be slightly improved in the losartan-treated group, this trend was not statistically significant (Figures 2B–D).

**FIGURE 3:**
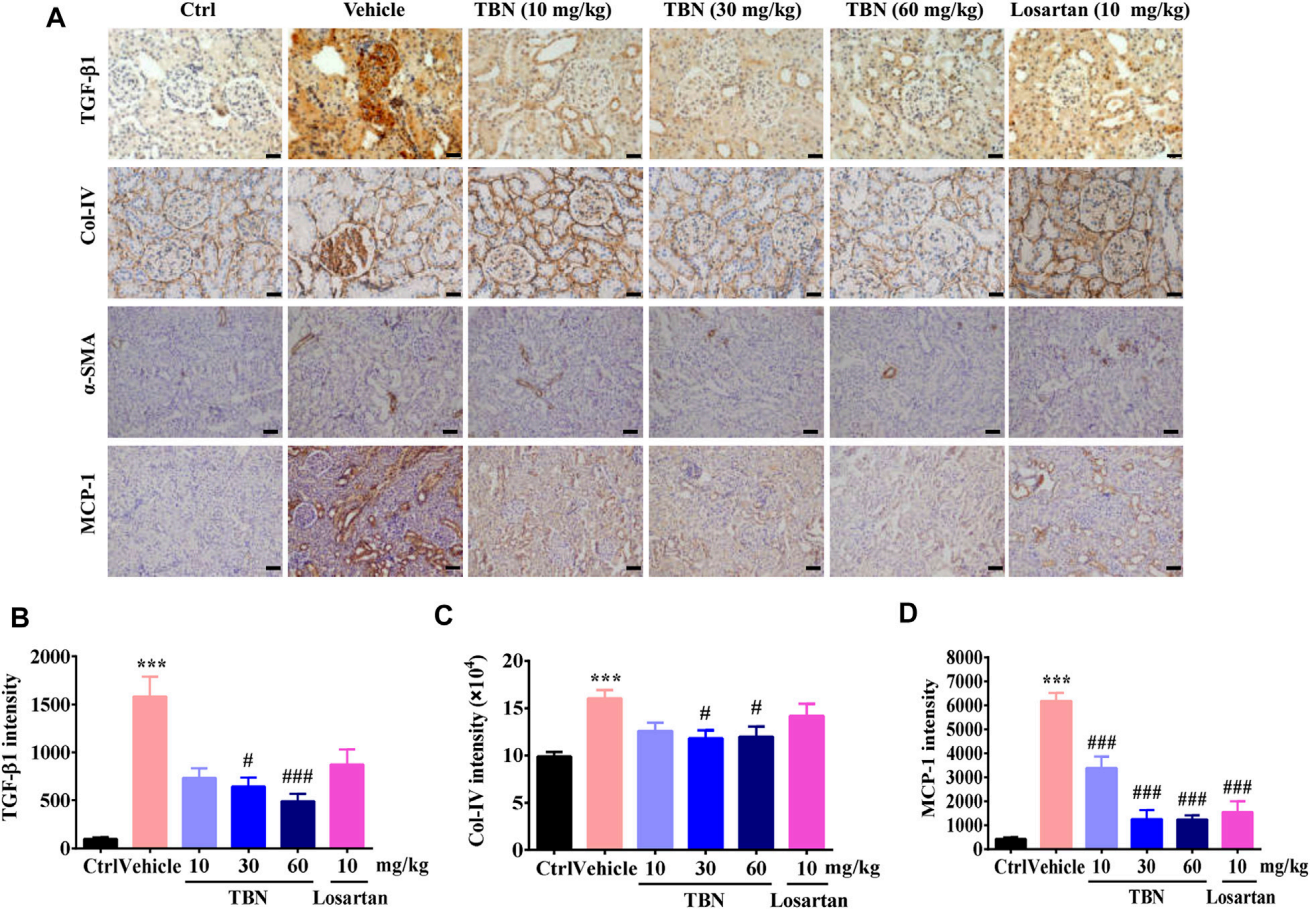
TBN Reduces fibrosis and interstitial inflammation in Renal Tissue of STZ-treated Rats. **(A)** Representative immunohistochemical images of TGF-β1, Col-IV, α-SMA, and MCP-1 staining. Immunohistochemistry staining of TGF-β1 and Col-IV was observed under 400× phase contrast microscope. Immunohistochemistry staining of α-SMA and MCP-1 was observed under 200× phase contrast microscope. **(B-D)** Semi-quantitative analysis of TGF-β1, Col-IV, and MCP-1 immunostaining intensity in the kidney for different groups were shown. Brown staining indicates the immunopositive area for the protein of interest. 30 images of immunostaining sections were counted for each rat. *n* = 13 for vehicle-treated DM group and *n* = 16 for other groups in TGF-β1 immunostaining. *n* = 8 for each group in Col-IV immunostaining. *n* = 5 for each group in MCP-1 immunostaining. Data were expressed as means ± SEM and were analyzed by 1-way ANOVA with Tukey’s test. ** *p* < 0.01 and *** *p* < 0.001 vs. Ctrl group. ^#^ *p* < 0.05, ^##^ *p* < 0.01, and ^###^ *p* < 0.001 vs. vehicle-treated group.

Renal tissue fibrosis was estimated by immunohistochemical staining of transformed growth factor-β1 (TGF-β1), type IV collagen (Col-IV), and alpha-smooth muscle actin (α-SMA, Figure 3A). Compared with the normal control group, positive staining of TGF-β1 and Col-IV was significantly increased in the renal cortex of vehicle-treated STZ rats. Renal cortex staining of TGF-β1 and Col-IV were significantly decreased by TBN treatment. However, losartan treatment had no effect on the expression of TGF-β1 and Col-IV (Figures 3B,C). Representative pictures of α-SMA staining were shown in Figure 3A. Positive α-SMA reaction were only observed in wall of blood vessels of control rats. Black arrowheads denote α-SMA-positive blood vessels. In the STZ-induced DKD rats, the expression of SMA was observed in glomerular mesangial (red arrowheads) and cytoplasm of tubular epithelial cells (green arrowheads), in addition to blood vessels (black arrowheads). However, there was expressed α-SMA in the wall of blood vessels (black arrowheads) and renal tubules (green arrowheads) without any expression in glomeruli by high-dose TBN treatment (Figure 3A). To confirm the interstitial inflammation, the expression of MCP-1 was examined by immunohistochemistry staining in renal tissues of STZ-induced DKD rats. Increased immunostaining of MCP-1 in the tubulointerstitial regions and weak staining in glomeruli were observed in STZ rats (Figure 3A). This result reveals that STZ increased the inflammatory response in kidney interstitial. Compared with the normal control group, positive staining of MCP-1 was significantly increased in the renal cortex of vehicle-treated STZ rats. However, the treatment with TBN and losartan could significantly reverse this increase (Figure 3D).

**FIGURE 4:**
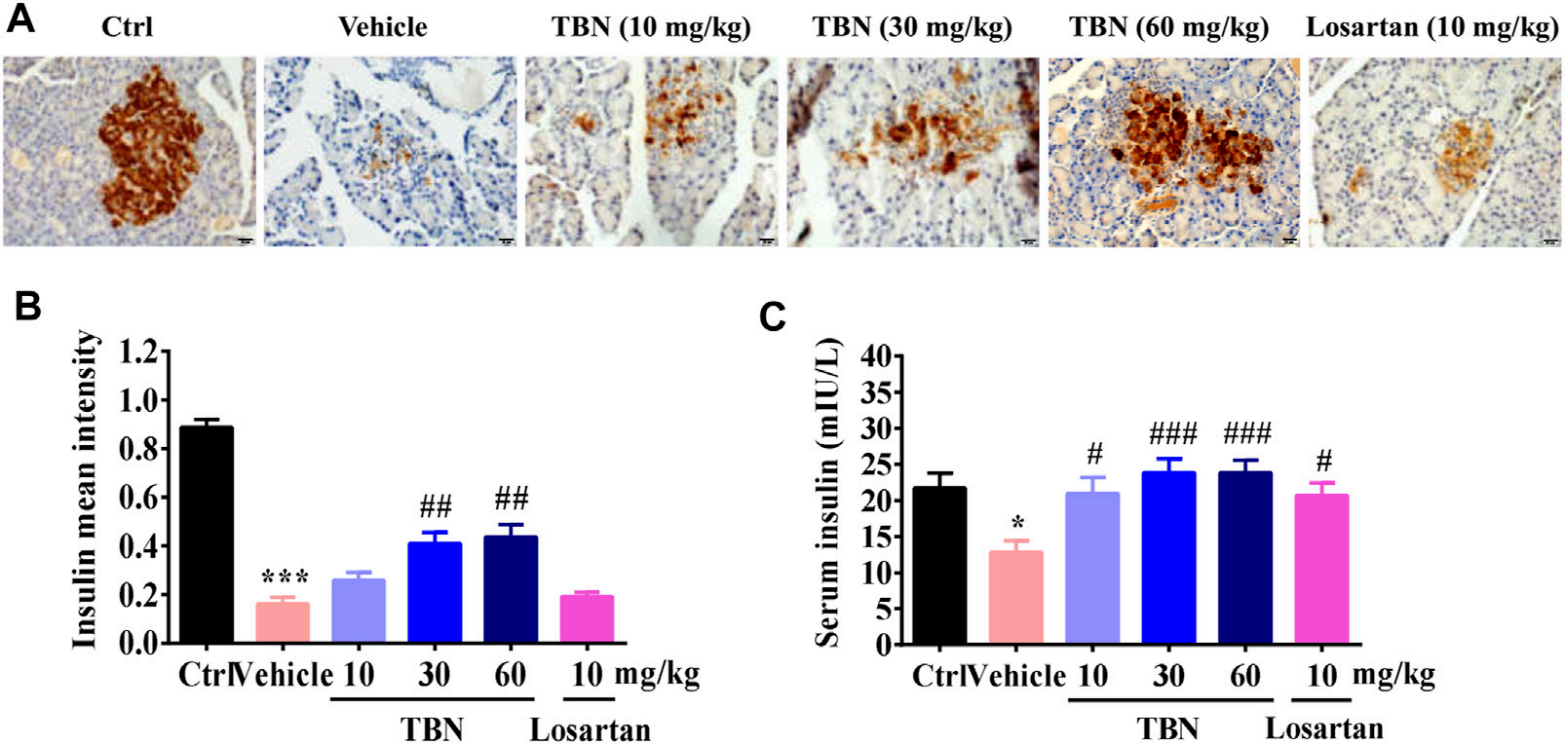
TBN protects islet beta cells and increases insulin secretion in STZ-induced rats. **(A)** Representative photomicrographs of insulin immunostaining in the pancreases of different groups. Scale bars, 20 µm. **(B)** Semi-quantitative analysis of insulin immunostaining in the pancreas. Brown staining indicates the immunopositive area for the protein of interest. 30 images of immunostaining sections were counted for each rat. *n* = 13 for vehicle-treated DM group and *n* = 16 for other groups. **(C)** Serum concentrations of insulin in STZ-induced DKD rats. Data were expressed as means ± SEM. Significance was determined by one-way ANOVA followed by tukey’s multiple comparisons test. *** *p* < 0.001 vs. Ctrl group. ^###^ *p* < 0.001 vs. vehicle-treated group.

To confirm that TBN treatment led to reduced blood glucose levels through protection of pancreatic tissue, thereby enabling the pancreas to secrete a more optimal amount of insulin, we analyzed the insulin levels in pancreatic tissue and sera. As expected, TBN treatment significantly protected the pancreas from the STZ injury (Figures 4A,B) and increased insulin levels in sera (Figure 4C). Losartan treatment had no obvious effect on insulin expression in the pancreatic tissue of DKD rats (Figures 4A,B).

### Tetramethylpyrazine Nitrone Alleviates Cataract Severity and Retinopathy in STZ-Induced DKD Rats

**FIGURE 5:**
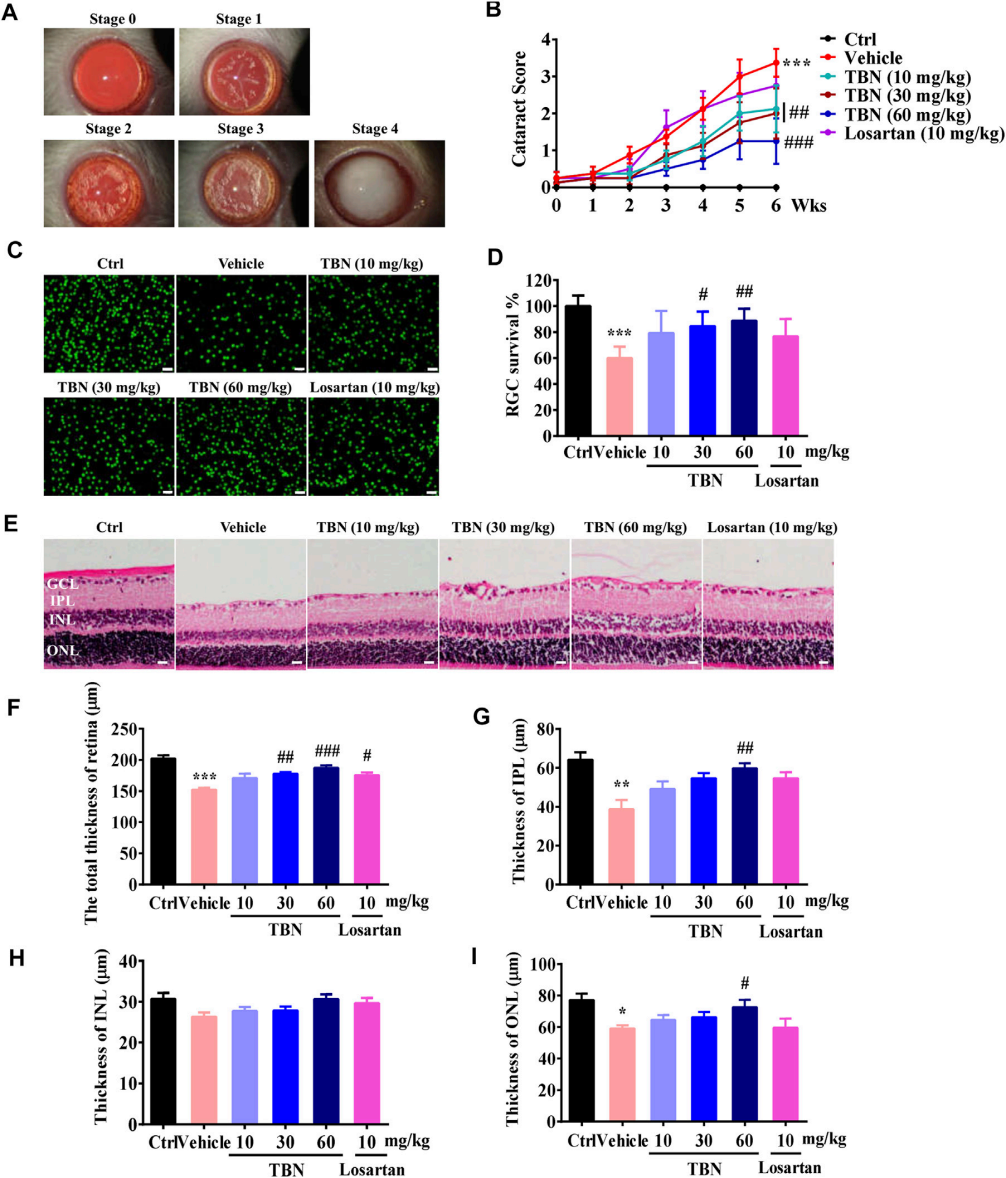
TBN alleviates cataract severity and retinal thickness reduction in STZ-induced rats. (**A)** Representative images of cataracts for stage scoring. (**B)** Cataracts were scored every week beginning at 3 weeks post-STZ injection. Data were means ± SEM of eight rats per group. (**C)** Representative immunofluorescence images of Brn3a staining in the retinas. Scale bars, 50 µm. (**D)** RGC survival rate in the different groups. (**E)** H&E staining of the retina. (**F-I)** Thickness of each retinal layer was measured. GCL, ganglion cell layer. IPL, internal plexiform layer. INL, internal nuclear layer. ONL, outer nuclear layer. Scale bar: 20 μm. All data were shown as the mean ± SEM of 5–6 rats per group. Data were expressed as mean ± SEM. Significance was determined by two-way ANOVA in figure B or one-way ANOVA followed by tukey’s multiple comparisons test. *p < 0.05, **p < 0.01 and ***p < 0.001 vs. Ctrl group. ^#^ *p* < 0.05, ^##^ *p* < 0.01, and ^###^ *p* < 0.001 vs. vehicle-treated group.

The kidney and eye share striking structural, developmental, physiologic, and pathogenic pathways, suggesting that kidney disease and ocular diseases may be similar in pathology (Nusinovici et al., 2019). Figure 5A shows representative images of cataracts from stages 0 to 4. Lenses of the normal control group appeared to be clear and normal (stage 0) during the experimental period. Vehicle-treated STZ rats had significantly increased cataract scores when compared with the normal control rats. Treatment with TBN markedly decreased the cataract scores of diabetic lenses. Losartan treatment, however, had marginal effect upon cataract scores (Figure 5B).

DKD and diabetic retinopathy (DR) are two major microvascular complications of diabetes. DKD often coexists with advanced DR, suggesting that they may share similar microvascular pathophysiology. It is generally accepted that kidney dysfunction is associated with the presence and severity of DR (Park et al., 2015; Park H. C. et al., 2019). Clinically, DR is one of the most important diagnostic criteria for DKD. Immunofluorescent staining of retinal tissue from vehicle-treated STZ rats showed a marked reduction in Brn3a-positive RGCs. Loss of RGCs in the GCL was significantly suppressed after TBN treatment. Losartan treatment had no obvious effect on RGCs in the GCL of DKD rats (Figures 5C,D). The thickness of each retinal layer was measured in paraffin sections after H&E staining (Figure 5E). The total retinal thickness, as well as the thickness of the IPL and ONL were all obviously decreased in the vehicle-treated STZ rats; the exception was the INL. However, the changes in total retinal thickness, IPL thickness, and ONL thickness were significantly attenuated by 6 weeks of treatment with TBN. Losartan treatment also clearly ameliorated the decreases in total retinal thickness, but had no effect on the thicknesses of the IPL, INL, and ONL (Figures 5F–I).

### Tetramethylpyrazine Nitrone Prevents the Progression of DKD in Rhesus Macaques

Nonhuman primates provide ideal animal models for studying the mechanisms underlying human DKD due to their similarities to humans at molecular, biochemical, and pathophysiological levels (Wang et al., 2014). TBN was administered at a dose of 30 mg/kg twice daily for 12 weeks according to the study protocol. At the end of the 12 week treatment, neither TBN nor vehicle-treated rhesus macaques experienced any differences in body weight (Table 1 and Supplemental Table 1). The mean percent change in glycosylated hemoglobin (HbAlc) levels decreased 5.78% in the TBN group, but not in the vehicle-treated group (0%). The eGFR increased from 48 at baseline to 53 ml/min/1.73 m^2^ (10.54% increase) in the TBN group, but increased only modestly in the vehicle-treated group. The mean plasma creatinine (Cr-P) decreased by 8.48% from baseline in the TBN group. The mean percentage change in urinary CysC was -5.94 and 2.39% in the TBN and vehicle groups, respectively. Blood lipid analysis revealed changes in the levels of TG, TC, and LDL in the rhesus macaques. After 12 weeks of treatment, TG decreased by 18.05% from baseline in the TBN group, but decreased only modestly in the vehicle-treated group. Meanwhile, mean TC and LDL increased 15.79 and 45.04% from baseline in the vehicle group, respectively. TC decreased by 2.09% from baseline, and LDL increased by 2.21% in the TBN group. Consistent with the data from STZ-induced DKD rats, treatment with TBN reduced systemic oxidative damage in rhesus macaques with DKD. Compared with the vehicle-treated group, the TBN-treated group had decreased levels of serum MDA, 3-NT, and 8-OHdG, in addition to increased GPx7 by the end of the experiment. The serum biochemical parameters of the rhesus macaques are summarized in Table 1 and Supplemental Table 2.

**TABLE 1:** TBN prevents the progression of DKD in rhesus macaques.

Parameters	Vehicle	TBN (30 mg/kg)
BW (kg)	1.02 ± 1.32	−0.14 ± 2.23
HbA1c (µmol/L)	0.00 ± 0.14	−0.26 ± 0.15
eGFR (ml/min/1.73 m^2^)	1.00 ± 4.24	5.00 ± 5.10
Cr-P (mg/dl)	−0.09 ± 0.14	−0.11 ± 0.08
CysC (mg/L)	0.04 ± 0.02	−0.10 ± 0.15
TG (mmol/L)	−0.01 ± 0.01	−0.18 ± 0.43
TC (mmol/L)	0.39 ± 0.26	−0.08 ± 0.18
LDL (mmol/L)	0.44 ± 0.20	0.03 ± 0.06
MDA (nmol/ml)	1.08 ± 1.12	−0.61 ± 1.02
GPx7 (nmol/ml)	−0.46 ± 2.72	−1.70 ± 17.88
3-NT (nmol/L)	−389.91 ± 421.04	−1,212.31 ± 241.19

Data presented are means ± SEM. Vehicle, vehicle-treated group, *n* = 2. TBN (30 mg/kg), TBN-treated group, *n* = 5.

### Tetramethylpyrazine Nitrone Increases Mitochondrial Function in HK-2 Cells

**FIGURE 6:**
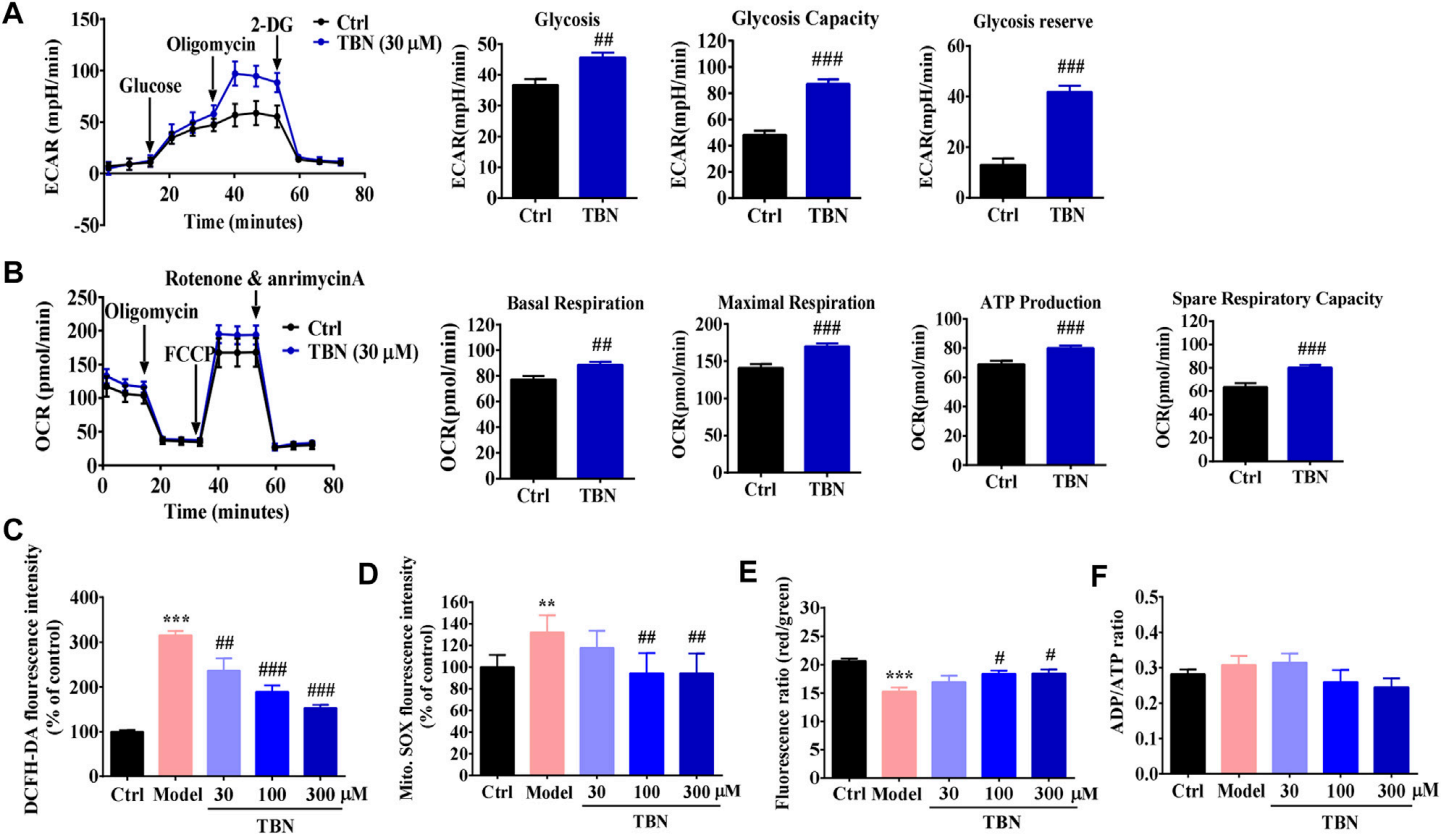
TBN improves mitochondrial function in HK-2 cells. **(A)** A glycolysis stress test was used to measure glycolytic function of HK-2 cells incubated with or without TBN. Representative graph of ECAR at baseline following sequential addition of 10 mM glucose, 1 µM oligomycin, and 50 mM 2-deoxyglucose (2-DG). Glycolysis, Glycolytic capacity, and Glycolytic reserve were calculated. (**B**) OCR were measured in HK-2 cells at baseline following sequential addition of 1 µM oligomycin, 0.5 µM carbonyl cyanide 4-trifluoromethoxy-phenylhydrazone (FCCP), and 0.5 µM each of rotenone and antimycin A. Basal respiration, Maximal respiration, ATP production, and Spare respiration capacity were calculated in HK-2 cells. For two-group comparisons, data were analyzed using an unpaired Student’s t-test. ^##^ *p* < 0.01 and ^###^ *p* < 0.001 vs. Ctrl. Cellular ROS production **(C)**, mitochondrial ROS production **(D)**, MMP **(E)**, and ATP generation **(F)** were analyzed in high glucose-induced HK-2 cells. Data represent mean ± SEM. Statistical significance was evaluated by 1-way ANOVA in J-M followed by Tukey’s tests. **p < 0.01 and ***p < 0.001 vs. ctrl group. ^#^ *p* < 0.05, ^##^ *p* < 0.01, and ^###^ *p* < 0.001 vs. model group.

Mitochondrial dysfunction is a key feature of DKD (Czajka et al., 2015). We next investigated the effects of TBN on glycolytic and mitochondrial respiration in HK-2 cells. Figure 6A shows the ECAR of HK-2 cells. Exposure to 30 μM TBN increased the ECAR of HK-2 cells responding to both glucose and oligomycin. In comparison to untreated HK-2 cells, TBN notably enhanced basal glycolysis and glycolytic capacity, as well as glycolytic reserve. As a measure of mitochondrial function, we assessed OCR in HK-2 cells (Figure 6B). Basal respiration, maximal mitochondrial respiration, ATP production, and spare respiration capacity were all significantly enhanced by TBN treatment. These results demonstrate that TBN significantly enhances glycolysis and mitochondrial function in HK-2 cells.

Mitochondrial changes seen in experimental and clinical models of DKD include increased mitochondrial ROS (mtROS) consequent to hyperglycemia, altered mitochondrial respiratory chain function, and damaged mitochondrial DNA and proteins (Qi et al., 2017). We next determined whether TBN could improve high glucose-induced mitochondrial dysfunction in HK-2 cells. While we observed a significant increase in intracellular and mtROS production in high glucose-induced HK-2 cells, we also found that TBN at concentrations of 30–300 μM significantly inhibited these high glucose-induced changes (Figures 6C,D). JC-1 staining also showed that TBN treatment inhibited the decrease in mitochondrial membrane potential (MMP) seen in high glucose-induced HK-2 cells (Figure 6E). We then determined energy generation in HK-2 cells as reflected by the ratio of ADP to ATP; however, no differences in the ADP/ATP ratio were observed (Figure 6F). These results from cultured HK-2 cells suggest that renoprotection of TBN may be linked to modulation of mitochondrial function.

### Tetramethylpyrazine Nitrone Improves Mitochondrial Function Through Activation of the AMPK/PGC-1α-Mediated Signaling Pathways

**FIGURE 7:**
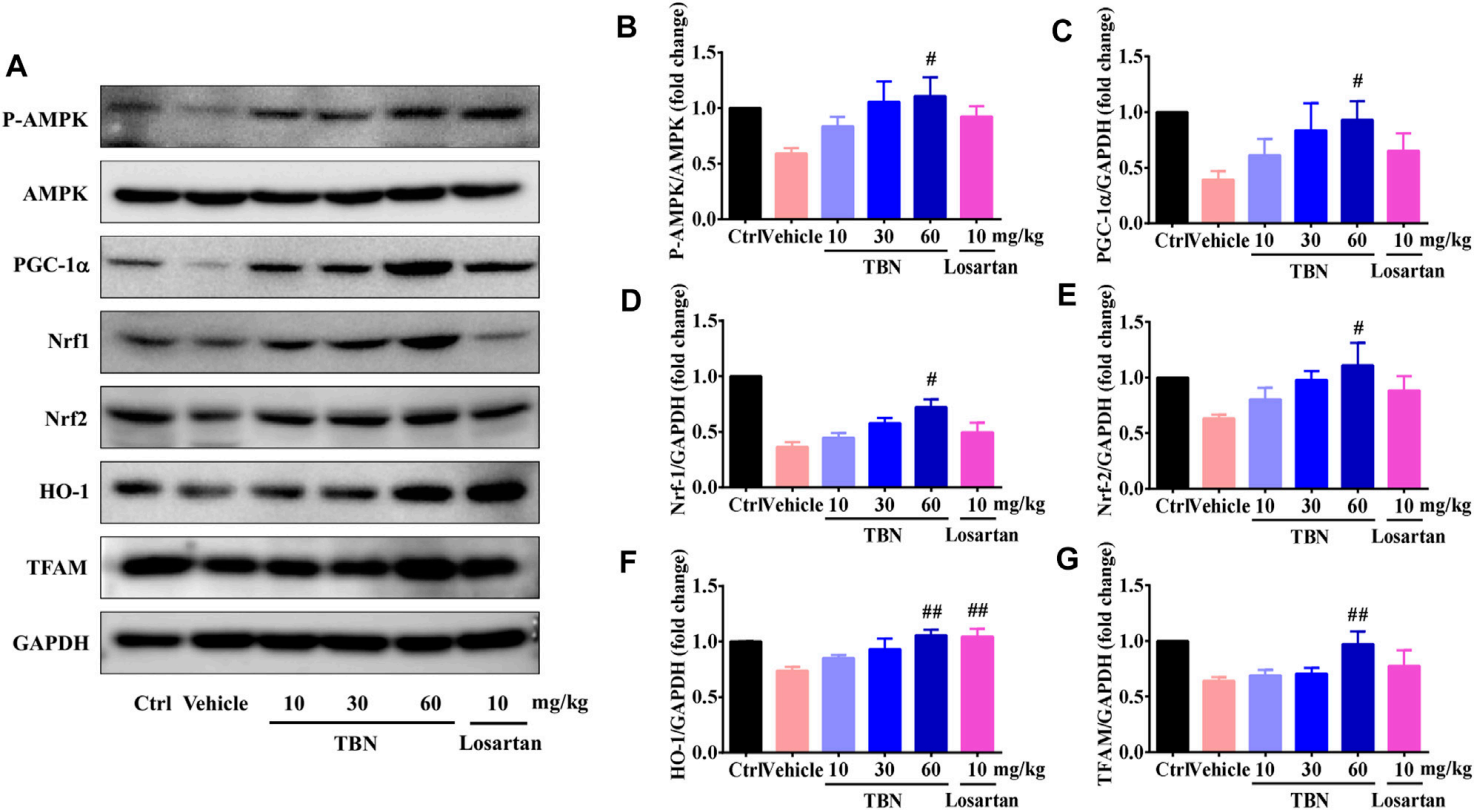
TBN upregulated *p*-AMPK, PGC-1α, Nrf1, Nrf2, HO-1, and TFAM expression in both STZ-induced rats. **(A)** Representative Western blots illustrating the expression of *p*-AMPK, total AMPK, PGC-1α, Nrf1, Nrf2, HO-1, and TFAM in the renal cortices of STZ-induced rats. Bar graphs showing mean Western blot of *p*-AMPK/AMPK (**B**), PGC-1α/GAPDH (**C**), Nrf1/GAPDH (**D**), Nrf2/GAPDH (**E**), HO-1/GAPDH (**F**), and TFAM/GAPDH (**G**) in the renal cortices of STZ-induced rats. Data are means ± SEM of six rats per group. Significance was determined by one-way ANOVA followed by Tukey’s test. *p < 0.05 and **p < 0.01 vs. Ctrl group. ^#^ *p* < 0.05 and ^##^ *p* < 0.01 vs. vehicle treated-STZ model group.

Upregulation of AMPK/PGC-1α can slow or halt the progression of DKD (Hong Y. A. et al., 2018). Given the profound effects of AMPK on mitochondrial function and redox-states, we tested whether TBN could activate AMPK, which could then mediate mitochondrial biogenesis and antioxidant defense by activating PGC-1α. Consistent with what was demonstrated in experimental models of DKD (Akhtar and Siragy, 2019), the expression of activated AMPK and PGC-1α were significantly lower in the renal cortices of vehicle-treated DKD rats when compared with that of the control rats. It was apparent that TBN treatment markedly increased the protein levels of activated AMPK and PGC-1α (Figures 7A–C). TBN treatment also promoted the expressions of Nrf1, Nrf2, and TFAM, which are the target genes of PGC-1α and executors of mitochondrial biogenesis (Figures 7D,E,G). Similarly, TBN also rescued the expression of HO-1, the controlling enzyme for oxidative stress. Losartan treatment, however, only significantly increased the expression of HO-1 in STZ-treated rats (Figure 7F). Taken together, these results indicate that the renal protection of TBN is associated with improved mitochondrial function and an enhanced cellular antioxidant defense system, and those improvements are achieved at least partially via regulating the AMPK/PGC-1α-mediated downstream signaling pathways.

## Discussion

DKD, a major microvascular complication of both type 1 and type 2 DM, is an important cause of end-stage renal disease. There are currently limited therapeutic options to slow or halt the progression of DKD. In this study, the measurements of biochemical parameters obtained in STZ rats and spontaneous DKD monkeys revealed that TBN can effectively ameliorate the deterioration of DKD. Urinary albumin, blood urea nitrogen, blood creatinine, and UACR are commonly used to identify renal dysfunction in clinical diagnoses of DKD. As shown in Figure 1, TBN treatment decreased urinary albumin, UACR levels, and blood urea nitrogen. In spontaneous DKD monkeys, TBN elevated eGFR and decreased CysC and Cr-P. Importantly, a strong correlation was found between the biochemical parameters and histopathological abnormalities in the rodent models. TBN treatment significantly reduced the glomerular hypertrophy, sclerotic lesions and fibrosis found in the rodent DKD models. Moreover, the significant elevations of blood glucose, blood triglyceride and cholesterol levels standard in DKD models were also reduced following TBN treatment. The mechanisms underlying TBN’s effectiveness are primarily due to its ability to reduce ROS, increase antioxidant defense capability, and enhance mitochondrial functions through activation of AMPK/PGC-1α-related signaling pathways.

In a non-clinical pharmacokinetics study, when SD rats were administered a single dose of TBN at 30 mg/kg intragastrically, the plasma concentrations of TBN were 56.51 μΜ and 64.44 μΜ, respectively, after 10 and 30 min of drug administration. At the same time, TBN concentrations in the kidney reached 46.72 and 55.08 μM (assuming the volume of 1 g kidney tissue is 1 ml), respectively (Supplemental Table 3). Our present pharmacokinetics and tissue distribution studies demonstrate that the effective concentration of TBN (above 30 μM) was achievable in both plasma and renal tissue when indicated doses of TBN were used in animal studies. Most importantly, in the phase I clinical study, when TBN was given to healthy human volunteers at 1,200 mg/person orally twice daily for seven consecutive days, its plasma C_max_ reached 114.35 μΜ (Supplemental Table 4). TBN showed significant efficacy in the STZ-induced DKD rat model at doses from 10–60 mg/kg; it is thus clear that the therapeutically effective plasma concentration of TBN in rats can be easily achieved in humans.

In the present study, TBN achieved better glycemic control than losartan did in STZ-induced DKD rats. A possible explanation could be that TBN ameliorates pancreatic tissue damage and thereby increased insulin secretion. IHC staining for insulin levels in STZ-treated rats confirmed this hypothesis. As expected, losartan had no significant effect in ameliorating pancreatic damage in DKD rats, consistent with a previous report that losartan treatment significantly helped all parameters of renal dysfunction except STZ-induced hypoinsulinemia (Murali et al., 2003). Furthermore, hyperglycemia is involved in increased diabetic renal growth (Wolf and Ziyadeh, 1999), which may explain why renal hypertrophy was attenuated to a greater extent by TBN than by losartan. The effect of TBN on diabetic hyperglycemia can thus potentially be attributed, at least in part, to its renoprotective properties.

It has been known for a long time that anemia is typically inevitable as kidney disease progresses, but the importance of treating the anemia secondary to renal disease has been undervalued in diabetic patients until recently. Although widely used in therapy for DKD, losartan can decrease hemoglobin levels and cause anemia, which is an independent risk factor in and of itself for chronic kidney disease progression (Mohanram et al., 2008). EPO deficiency and functional iron deficiency are two key causes underlying the development of anemia in chronic kidney disease (Gupta and Wish, 2017). In line with these studies, we found that EPO production and iron levels were markedly decreased in the sera of STZ-induced DKD rats; losartan-treated rats had even lower EPO levels than did vehicle-treated DKD rats. By contrast, TBN increased the levels of EPO and iron in DKD rat models. Taken together, we speculate that TBN might be also beneficial for the treatment of anemia in DKD patients.

Diabetic eye diseases, including diabetic cataracts and DR, are also among the most common complications related to small-vessel injuries resultant of long-term hyperglycemia (Sivaprasad et al., 2012). In fact, DR precedes the onset of overt nephropathy; that is, patients with proteinuria or patients who ultimately need dialysis frequently present first with vision-threatening DR (Boelter et al., 2006). As shown in Figure 5, TBN alleviated cataract severity and RGC loss while increasing retinal layer thickness in STZ-induced rats, and was more effective than losartan in doing so. Together with our previous finding that TBN significantly increased the survival of RGCs after N-methyl-D-aspartate insult in SD rats (Luo et al., 2017), these results suggest that TBN might also be beneficial in treating diabetic eye diseases.

In the past few decades, many therapies have been tested in animal models and have shown efficacy in treating DKD; however, translating these experimental therapies into viable clinical treatments is enormously challenging (Fernandez-Fernandez et al., 2014; Khan and Quaggin, 2015). One of the important reasons for the difficulty in translating experimental benefits to the clinic is that DKD animal models are different from actual diseased patients. Classical models of type 1 (STZ-induced rats and non-obese diabetic mice) or type 2 diabetes (*db/db* mice) only cause modest albuminuria without decreased GFR (Soler et al., 2012; Betz and Conway, 2014). Therapeutic interventions reported to reduce albuminuria and improve renal pathology in these models (e.g. RAAS blockade, statins, vitamin D) have, with the exception of RAAS targeting, produced negative results when tested in humans (Deb et al., 2010).

Nonhuman primate models of type 2 diabetes are excellent for investigating new therapeutic approaches, particularly when it comes to evaluating mechanisms of action and assessing therapies’ potential application to humans (Young et al., 1999). The complications of type 2 diabetes typically seen in humans, including nephropathy, have also been identified in nonhuman primates (Hansen et al., 2011). In our study, rhesus monkeys aged 14–22 years with type 2 diabetes were diagnosed with stage III DKD according to the following criteria: eGFR 30–59 ml/min/1.73 m^2^, CysC ≥1.3 mg/dl, and Scr ≥1.26 mg/dl. Encouragingly, 12 weeks of treatment with TBN elevated the monkeys’ eGFR and reduced CysC. Similarly, TBN improved their abnormal glucose and lipid metabolism in STZ-treated rats. TBN also reduced lipid peroxidation, DNA, and protein oxidative damage, while also increasing antioxidant defense. The data from this nonhuman primate model provides solid support for development of TBN as a nephroprotective agent for patients with DKD.

High levels of ROS accumulation was found in both animal models of DKD as well as human DKD patients, suggesting that ROS contribute to the development and exacerbation of DKD (Zhan et al., 2015; Galvan et al., 2017). We previously reported that TBN directly scavenges O2·^–^, ·OH, and ONOO^−^ (Sun et al., 2012). Moreover, our recent research demonstrates that TBN at concentrations from 30–300 μM significantly decreased the levels of these free radicals in primary cortical neurons as well as in isolated cortical mitochondria; TBN also increased MMP and decreased mitochondrial swelling (Zhang et al., 2018). In the current study, we assessed whether TBN has beneficial effects against hyperglycemia-induced systemic and renal oxidative stress by measuring serum MDA and urinary 8-OHdG levels. The present findings are consistent with our previous reports. TBN treatment lowered urinary 8-OHdG levels in STZ-treated rats. Compared with vehicle treatment, TBN decreased the levels of MDA, 3-NT, and 8-OHdG in rhesus macaques with autonomic DKD. The mitochondria are a principal source of ROS production, and conversely, it is especially susceptible to damage from ROS (Park C. et al., 2019). Thus, we also assessed mitochondrial oxidative stress by measuring accumulation of mtROS in high glucose-induced HK-2 cells. In the *in vitro* study, we confirmed that TBN at a concentration range of 30–300 μM significantly inhibited hyperglycemia-induced intracellular and mtROS production. Taken together, these results indicate that TBN improves DKD, at least in part, by reducing the hyperglycemia-induced systemic, renal, and particularly mitochondrial oxidative stress seen in DKD.

Mitochondria are complex organelles that regulate a variety of cellular processes and functions, including redox regulation, cytosolic calcium and apoptosis. Most importantly, mitochondria are the major sites of cellular ATP production (Bhargava and Schnellmann, 2017). In general, all cell types in the kidney need ATP to maintain cellular functions. Proximal tubules in particular depend on the efficiency of mitochondrial oxidative phosphorylation to produce the ATP needed to drive the active transport of glucose, ions, and nutrients (Weinberg et al., 2000). Previous studies have shown that the increased rates of renal ROS production seen in diabetes damages cellular components such as mitochondrial DNA, proteins, and lipids. This process impairs mitochondrial function and reduces ATP production (Ruiz et al., 2013). Indeed, both clinical and experimental DKD are characterized by mitochondrial dysfunction (Sharma et al., 2013; Coughlan et al., 2016). Our data further validate prior findings TBN ameliorates high glucose-induced excessive accumulation of mtROS, ATP depletion, and MMP reduction in HK-2 cells.

**FIGURE 8:**
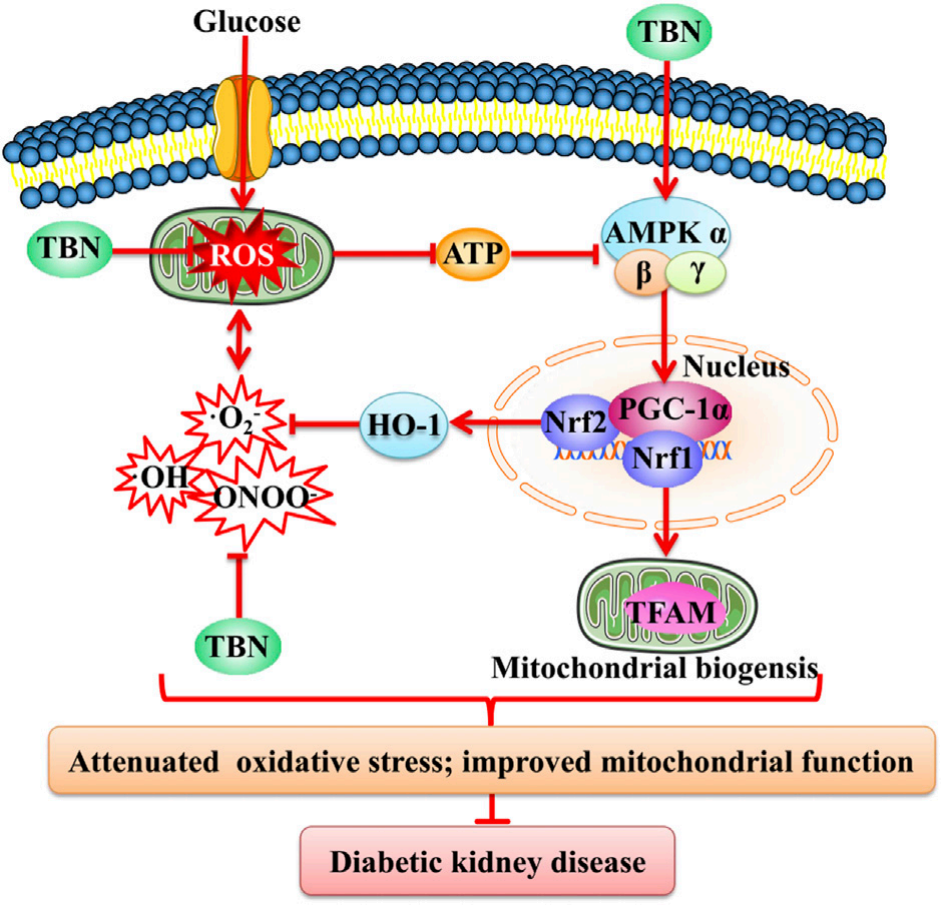
Schematic representation of the mechanism of TBN against DKD. TBN improved mitochondrial biogenesis and renal function by inhibiting oxidative stress. HG, high glucose; ATP, adenosine triphosphate; AMPK, adenosine monophosphate activated protein kinase; PGC-1α, peroxisome proliferator-activated receptor-gamma coactivator-1α; HO-1, heme oxygenase-1; Nrf2, nuclear factor erythroid-2-related factor 2; 8-OHdG, 8-hydroxy-2′-deoxyguanosine.

Reduced AMPK activity may be a key molecular mechanism causing mitochondrial dysfunction in diabetic kidneys. AMPK is a cellular energy sensor that acts to preserve cell survival under conditions of low substrate availability. AMPK activation promotes mitochondrial substrate utilization and ATP generation in parallel with the maintenance of intracellular redox balance (Hardie et al., 2012). In diabetes, renal inactivation of AMPK is known to be associated with impaired mitochondrial function (Dugan et al., 2013). PGC-1α is the major co-activator needed to regulate mitochondrial biogenesis; reduced levels of PGC-1α have been observed in diabetic rat kidneys (Guo et al., 2015). AMPK activation improves DKD by increasing PGC-1α-regulated mitochondrial biogenesis and the activity of nuclear factor Nrf2-induced downstream antioxidant enzymes like HO-1. This suppresses oxidative stress and thus constitutes an energy sensing network that controls energy expenditure, including mitochondrial energy metabolism (Canto and Auwerx, 2009; Zheng et al., 2011). Upregulation of AMPK/PGC-1α-mediated downstream signaling pathways is hence predicted to possibly slow or halt the progression of DKD. TBN promotes AMPK activation and increases the expression of PGC-1α, Nrf1, Nrf2, HO-1, and TFAM. In HK-2 cells, TBN increased glycolysis and mitochondrial respiration rates. Our study suggests that TBN improves mitochondrial dysfunction and suppresses oxidative stress to slow the progression of DKD, possibly through upregulation of AMPK/PGC-1α-mediated downstream signaling pathways (Figure 8).

In summary, our current study demonstrates that TBN protected against DKD in STZ-induced rats and non-human primates by suppressing oxidative stress and improving mitochondrial function, possibly via activation of the AMPK/PGC-1α-related signaling pathway. Our preliminary data also showed that TBN was beneficial for treating diabetic eye diseases and DKD anemia. Based on its superior therapeutic efficacy in animal models, novel mechanisms of action, and excellent safety profile as seen in both animals and a phase I human study, we are currently investigating TBN’s effect on DKD in a phase II clinical study.

## Data Availability

The original contributions presented in the study are included in the article/Supplementary Material, further inquiries can be directed to the corresponding authors.
