# Supplementary material for: Nephroprotective Effects of Tetramethylpyrazine Nitrone TBN in Diabetic Kidney Disease
Source: Front Pharmacol. 2021 Jun 24;12:680336. doi: 10.3389/fphar.2021.680336 (PMC8264657; doi:10.3389/fphar.2021.680336)
Supplement: Supplementary file 1 [file DataSheet1.PDF]

Supplementary Material

Supplementary Figure 1. The chemical structures of TMP, NXY-059, and TBN.

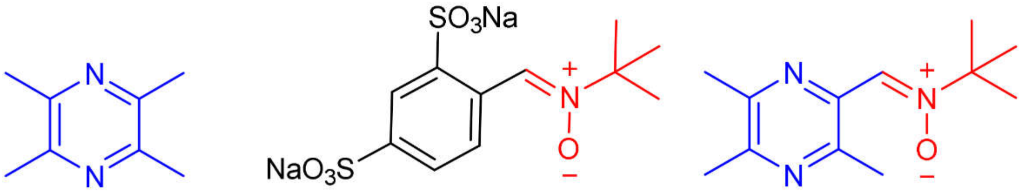

Tetramethylpyrazine (TMP) **Disufenton sodium (NXY-059)** **Tetramethylpyrazine nitrone (TBN)**

Supplementary Table 1

Table 1. The body weights of rhesus macaques.

Animals	V1	V2	T1	T2	T3	T4	T5
Baseline	8.96	10.44	10.69	10.51	13.73	12.07	12.46
	9.70 ± 1.05		11.61 ± 0.99				
Wk1	8.99	10.63	10.78	10.54	13.48	11.76	12.24
	9.81 ± 1.16		11.62 ± 1.01				
Wk2	9.19	10.69	10.95	10.65	13.38	11.61	11.97
	9.94 ± 1.06		11.94 ± 1.04				
Wk3	9.36	10.79	10.88	10.71	13.19	11.4	11.85
	10.08 ± 1.01		11.45 ± 0.84				
Wk4	9.44	10.91	10.95	10.81	13.32	11.39	11.65
	10.18 ± 1.04		11.63 ± 0.95				
Wk5	9.78	10.8	11.24	11.31	13.69	11.35	12.12
	10.29 ± 0.72		11.76 ± 1.08				
Wk6	9.54	10.33	10.96	10.9	12.89	11.03	11.45
	9.94 ± 0.56		11.75 ± 1.13				
Wk7	9.98	10.56	11.34	11.03	13.28	10.95	11.57
	10.27 ± 0.41		11.73 ± 1.12				
Wk8	10.12	10.62	11.63	11.01	13.54	10.8	11.8
	10.37 ± 0.35		11.61 ± 1.02				

Wk9	10.26	10.46	11.79	11.01	13.56	10.62	11.75
	10.36 ± 0.14		11.75 ± 1.21				
Wk10	10.49	10.52	11.82	11.24	13.5	10.44	11.64
	10.51 ± 0.02		11.61 ± 0.99				
Wk11	10.68	10.42	11.66	11.14	13.23	10.45	11.57
	10.55 ± 0.18		11.62 ± 1.01				
Wk12	10.91	10.53	11.87	13.51	11.36	10.15	11.88
	10.72 ± 0.27		11.94 ± 1.04				

Notes: V, vehicle-treated. T, TBN-treated. Wk, week.

Supplementary Table 2

Table 2 The raw data of rhesus macaques.

Animals		V1	V2	T1	T2	T3	T4	T5
	Wks/Age(yrs.)	14	22	16	15	18	16	16
HbA1c (μmol/L)	0	4.50	4.80	4.70	4.70	4.60	4.90	4.90
	4	4.50	4.80	4.60	4.70	4.60	4.80	4.80
	8	4.60	4.90	4.40	4.40	4.60	4.50	4.50
	12	4.60	4.70	4.50	4.60	4.30	4.70	4.40
	Change (0-12 Wks)	0.00%		-5.78%				
eGFR ($ml/min/1.73m^2$)	0	54.00	45.00	55.00	43.00	46.00	50.00	46.00
	4	55.00	45.00	54.00	45.00	54.00	55.00	42.00
	8	53.00	47.00	61.00	51.00	56.00	57.00	49.00
	12	52.00	49.00	58.00	49.00	58.00	56.00	44.00
	Change (0-12 Wks)	2.58%		10.54%				
Cr-P (mg/dL)	0	1.48	1.43	1.28	1.48	1.49	1.29	1.40
	4	1.49	1.29	1.20	1.39	1.27	1.12	1.47
	8	1.51	1.39	1.11	1.33	1.25	1.12	1.31
	12	1.49	1.24	1.18	1.35	1.30	1.14	1.42
	Change (0-12 Wks)	-6.30%		-8.48%				

CysC (mg/L)	0	1.43	1.57	1.49	1.93	1.61	1.71	1.77
	4	1.39	1.76	1.62	1.87	1.49	1.69	1.94
	8	1.42	1.54	1.46	1.46	1.41	1.60	1.73
	12	1.48	1.59	1.48	1.72	1.33	1.61	1.86
	Change (0-12 Wks)	2.39%		-5.94%				
TG (mmol/L)	0	0.50	0.51	0.41	0.60	0.85	1.21	0.65
	4	0.49	0.56	0.38	0.59	0.65	0.45	0.56
	8	0.45	0.62	0.57	0.54	0.73	0.36	0.57
	12	0.49	0.51	0.27	0.49	0.81	0.32	0.91
	Change (0-12 Wks)	-1.00%		-18.05%				
TC (mmol/L)	0	2.35	2.47	2.79	2.62	3.33	3.10	3.60
	4	2.51	3.06	2.85	2.45	3.04	3.01	3.40
	8	2.63	3.31	2.87	2.83	3.32	3.43	3.68
	12	2.55	3.04	2.76	2.73	3.17	3.13	3.25
	Change (0-12 Wks)	15.79%		-2.09%				
LDL (mmol/L)	0	0.67	1.28	1.63	1.40	1.21	1.04	1.55
	4	0.82	1.66	1.36	1.43	1.09	0.93	1.60
	8	0.95	1.84	1.81	1.51	1.41	1.23	1.59
	12	0.97	1.86	1.62	1.41	1.35	1.04	1.54

	Change (0-12 Wks)	45.04%		2.21%				
MDA (nmol/ml)	0	2.61	0.76	2.22	1.23	4.70	4.14	2.90
	4	1.51	1.08	2.32	2.32	2.33	0.15	0.99
	8	3.33	1.58	3.07	2.29	2.68	0.44	0.37
	12	2.89	2.63	2.33	1.81	2.82	2.76	2.41
	Change (0-12 Wks)	128.39%		-7.62%				
GPx7 (nmol/ml)	4	10.71	4.41	35.62	11.77	8.48	9.00	7.14
	8	14.29	1.47	34.25	8.82	18.64	3.00	7.14
	12	8.33	5.88	16.44	35.29	18.64	-4.00	-2.86
	Change (0-12 Wks)	6.94%		12.74%				
3-NT (nmol/L)	0	4450.96	4308.82	4661.60	4636.63	4728.18	4831.90	4414.46
	4	4203.18	4173.73	4402.94	3774.22	4202.54	4180.13	3900.35
	8	4021.35	4183.97	4296.66	4050.16	3921.47	4168.61	4392.05
	12	3763.33	4216.63	3808.79	3172.38	3443.53	3471.38	3315.16
	Change (0-12 Wks)	-8.80%		-26.02%				
8-OHdG (ng/mL)	0	8.18	10.17	17.63	12.80	8.25	10.53	9.76
	4	7.93	10.65	14.89	5.42	8.02	9.01	6.78
	8	8.25	7.09	10.61	7.31	7.48	8.68	8.47

	12	7.76	8.23	14.47	5.69	8.77	9.29	6.77
	Change (0-12 Wks)	-12.11%		-21.92%				

Notes: V, vehicle-treated. T, TBN-treated. Wks, weeks.

Supplementary Table 3

Table 3: TBN concentration in the plasma and kidney of rats after a single 30 mg/kg intragastric administration.

Parameters	TBN	
	10 min	30 min
Concentration in plasma (μM)	56.51	64.44
Concentration in the kidney (μM)	46.72	55.08

Supplementary Table 4

Table 4: Pharmacokinetic Parameters of TBN tablets in healthy Chinese volunteers after multiple-dose administration for 7 days.

**Parameter**	**1200 mg**
$t_{1/2}$ (h)	2.30
T_{max} (h)	2.56
C_{max} (μM)	114.35
C_{min} (μM)	2.37
C_{avg} (ng/ml)	6775.17
CLss/F (ng/ml)	17397.63
Vss/F (mL/h)	57710.15
$AUC_{0\text{-}tau}$ (h·ng/ml)	81302.08

$\mathbf{t_{1/2}}$ = elimination half-life; $\mathbf{T_{max}}$ = time to peak concentration; $\mathbf{C_{max}}$ = maximum serum concentration; $\mathbf{C_{min}}$ = steady state trough concentration; **Cavg** = average steady state concentration; **Clss/F** = steady state clearance; **Vss/F** = steady state volume of distribution; $\mathbf{AUC_{0\text{-}tau}}$ = AUC at steady state during one dose interval.
